# Dynamic derangement in amino acid profile during and after a stroke-like episode in adult-onset mitochondrial disease: a case report

**DOI:** 10.1186/s13256-019-2255-9

**Published:** 2019-10-21

**Authors:** Mai Fukuda, Yoshiro Nagao

**Affiliations:** 1 Hidaka Tokushukai Hospital, 1-10-27 Shizunai Kose-cho, Shin-Hidaka-cho, Hokkaido 056-0005 Japan; 2 Present Address: Fukuoka Tokushukai Hospital, 4-5 Sugukita, Kasuga city, Fukuoka 816-0864 Japan

**Keywords:** Maternally inherited diabetes and deafness, Mitochondrial myopathy, encephalopathy, lactic acidosis, and stroke-like episodes, Growth differentiation factor-15, Arginine, Citrulline, Aspartic acid, Taurine, Case report

## Abstract

**Background:**

Maternally inherited diabetes and deafness, and mitochondrial myopathy, encephalopathy, lactic acidosis, and stroke-like episodes are examples of mitochondrial diseases that are relatively common in the adult population. Mitochondrial myopathy, encephalopathy, lactic acidosis, and stroke-like episodes are assumed to be associated with decreases in arginine and citrulline. Biomarkers, such as growth differentiation factor-15, were developed to assist in the diagnosis of mitochondrial diseases.

**Case presentation:**

A 55-year-old Japanese man, an insulin user, presented after a loss of consciousness. A laboratory test showed diabetic ketoacidosis. He and his mother had severe hearing difficulty. Bilateral lesions on magnetic resonance imaging, the presence of seizure, and an elevated ratio of lactate to pyruvate, altogether suggested a diagnosis of mitochondrial disease. Mitochondrial DNA in our patient’s peripheral blood was positive with a 3243A>G mutation, which is the most frequent cause of maternally inherited diabetes and deafness, and mitochondrial myopathy, encephalopathy, lactic acidosis, and stroke-like episodes. As a result, maternally inherited diabetes and deafness/mitochondrial myopathy, encephalopathy, lactic acidosis, and stroke-like episodes was diagnosed. We measured growth differentiation factor-15 and multiple amino acids in his blood, longitudinally during and after the stroke-like episode. Growth differentiation factor-15 was increased to an immeasurably high level on the day of the stroke-like episode. Although his diabetes improved with an increased dose of insulin, the growth differentiation factor-15 level gradually increased, suggesting that his mitochondrial insufficiency did not improve. Multiple amino acid species, including arginine, citrulline, and taurine, showed a decreased level on the day of the episode and a sharp increase the next day. In contrast, the level of aspartic acid increased to an extremely high level on the day of the episode, and decreased gradually thereafter.

**Conclusions:**

Growth differentiation factor-15 can be used not only for the diagnosis of mitochondrial disease, but as an indicator of its acute exacerbation. A stroke-like episode of mitochondrial myopathy, encephalopathy, lactic acidosis, and stroke-like episodes reflects a drastic derangement of multiple amino acids. The involvement of aspartic acid in the episodes should be explored in future studies.

## Background

Mitochondrial disease is not a rare cause of diabetes mellitus in the adult population. At least 1% of individuals with diabetes in Japan are affected by maternally inherited diabetes and deafness (MIDD) [1, 2], which is a subtype of mitochondrial disease. Adult mitochondrial diseases may be disguised as other diseases, and hence are not diagnosed as such [3]. Genetic testing is not sensitive [4]. Therefore, biomarkers of mitochondrial disease are developed. Among the biomarkers, the lactate-to-pyruvate ratio is most sensitive and specific, next to growth differentiation factor (GDF)-15 [2, 5, 6]. Lactate, however, can be affected by medications for diabetes. In contrast, the treatment of diabetes does not interfere with GDF-15 [7].

Treatment of mitochondrial disease is emerging as well [6]. Acute exacerbations of mitochondrial myopathy, encephalopathy, lactic acidosis, and stroke-like episodes (MELAS) were associated with a decrease in arginine [8–11] and citrulline [12]. Based on these findings, intravenously and orally administered L-arginine are recommended as the standard treatment for MELAS [13–15]. It is hypothesized that L-arginine provides the substrate for nitric oxide synthase, and hence ameliorates the vasoconstriction in MELAS [16]. On the other hand, taurine modification of a mitochondrial transfer RNA was found to be deficient in MELAS [17]. Moreover, supplementation with taurine was preventive against stroke-like episodes of MELAS [18].

We describe a patient with MIDD/MELAS to whom these new modes of diagnosis and treatment (that is, GDF-15 and L-arginine) were applied. We also obtained unusual findings from longitudinal measurements of multiple amino acids during the stroke-like episode of MELAS and the follow-up period.

## Case presentation

**Fig. 1:**
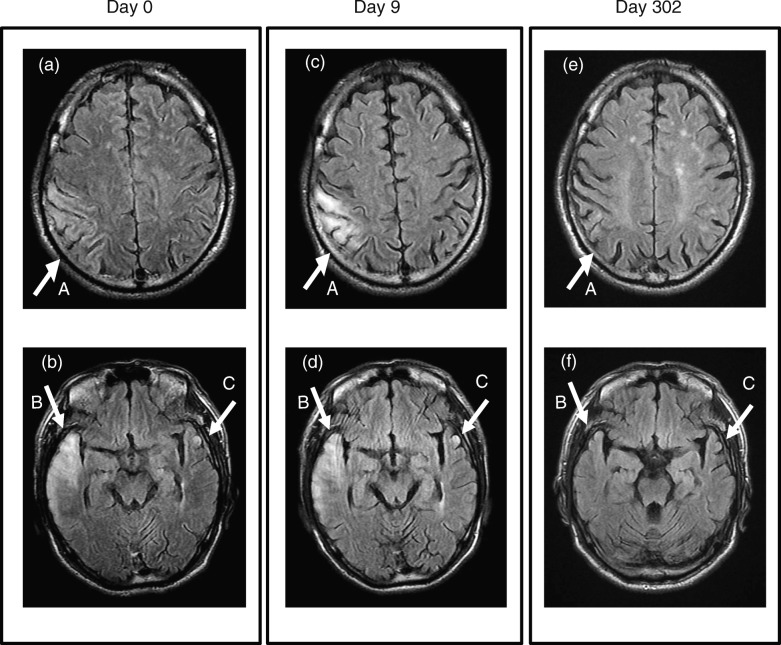
Magnetic resonance imaging of the brain. Fluid-attenuated inversion recovery (FLAIR) showed lesions in the right parietal lobe (pointed by the arrow A), right temporal lobe (arrow B), and left temporal lobe (arrow C) on day 0 (**a**, **b**). The lesion on the right parietal lobe (arrow A) intensified on day 9 (**c**, **d**). All of these lesions diminished by day 302 (**e**, **f**)

A 55-year-old Japanese man with diabetes presented with a loss of consciousness and bilateral convulsion (day 0) in 2017. His left upper and lower limbs were paralyzed and did not respond to painful stimuli. He was afebrile (36.8 °C), hypertensive (142/92 mmHg), and tachycardiac (108/minute). Diabetic ketoacidosis (DKA) was diagnosed, based on an acidosis (pH 6.95) and only moderately elevated blood glucose (486 mg/dL or 27 mmol/L). With the standard treatment for DKA (that is, fluid resuscitation, continuous infusion of insulin, and supplementation of potassium), he recovered consciousness 12 hours after the onset of illness. Left-sided hemiparesis diminished by day 1. We considered cerebral infarction as the most likely differential diagnosis, and conducted a magnetic resonance imaging (MRI) study. Fluid-attenuated inversion recovery (FLAIR) showed bilateral lesions (that is, right temporal lobe, right parietal lobe, and left temporal lobe), which were not consistent with vascular territory (Fig. 1a, b). The most affected lesion moved from the right temporal lobe (day 0) to the right parietal lobe by day 9 (Fig. 1c, d). These bilateral, more or less migratory, findings in FLAIR were compatible with mitochondrial disease, rather than with cerebral infarction [19–21]. Hyperglycemic encephalopathy, another differential diagnosis, was also unlikely considering his only moderately elevated blood glucose level. The lactate-to-pyruvate ratio (reference value, < 15) was repeatedly greater than 20. It had been reported that convulsion was rare in DKA, but was frequently associated with metabolic encephalopathy, especially of mitochondrial disease [22, 23]. Collectively, the imaging study, laboratory data, and clinical picture pointed to MELAS as the most likely diagnosis.

Our patient worked for an electronics store, and did not report a previous exposure to any toxic substance. He did not smoke tobacco or consume alcohol. He was thin (body mass index, 16, with 43 kg and 165 cm). His postprandial C-peptide-to-glucose ratio was low at repeated measurements (mean, 0.21 × 10^− 2^ ng/mg or 1.26 × 10^− 2^ nmol/mmol), indicating that his diabetes was due to impaired secretion of insulin [24, 25]. He had developed marginal diabetes mellitus in 2004, at 40 years of age, with glycated hemoglobin (HbA1c) of 6.6%. The next year, in 2005, he experienced his first episode of DKA, when his HbA1c was 12.6%. He had been treated with glargine (18 units/day), lispro (8 units/day), and glimepiride (0.5 mg/day), until the episode reported here. He noticed hearing difficulty at the age of 49 years, and began to wear a hearing aid at 50 years of age. Remarkably, his mother, a diabetic, lost her hearing at approximately the same age. This family history strongly suggested MIDD.

A genetic test of his peripheral blood was conducted at a commercial institution (SRL, Tokyo). This test showed that mitochondrial DNA was positive for the mutation of 3243A>G, which is the most frequent etiology for both MIDD and MELAS [26–29]. MIDD and MELAS are frequently overlapped in adults [30]. As a result, a diagnosis of MIDD overlapped with MELAS was entertained [23]. Our patient was discharged on day 22, when his diabetes was managed with glimepiride (0.5 mg/day), linagliptin (5 mg/day), glargine (18 units/day), and lispro (8 units/day). He was also prescribed to take aspirin (100 mg/day) and rosuvastatin (10 mg/day).

**Fig. 2:**
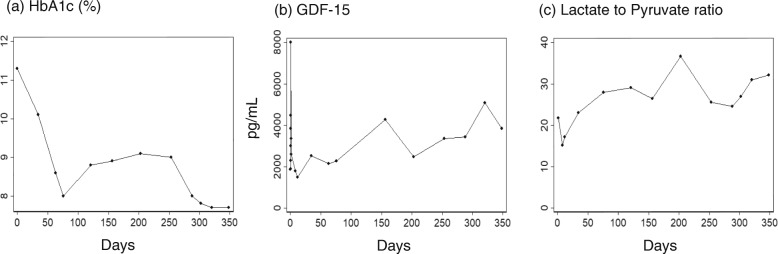
Severity of diabetes mellitus and mitochondrial disease. The severity of diabetes mellitus was represented with glycated hemoglobin (**a**). The severity of the mitochondrial disease was assessed with growth differentiation factor-15 (**b**) and lactate-to-pyruvate ratio (**c**). The values are plotted along the days that elapsed from the onset of the stroke-like episode. The reference value for growth differentiation factor-15 is below 707 pg/mL and that for lactate-to-pyruvate ratio is below 15. *HbA1c* glycated hemoglobin, *GDF-15* growth differentiation factor-15

After the discharge, we conducted a laboratory test (including HbA1c) once a month, and adjusted the dose of insulins. We ceased glimepiride on day 76, after which his HbA1c worsened (Fig. 2a). However, as we increased the dose of insulin, HbA1c improved, particularly after day 253. From day 253, we started treatment targeted at mitochondrial disease, with L-arginine (12 g/day), as well as the cocktail therapy for mitochondrial disease, which included L-carnitine (750 mg/day), Ubidecarenone (coenzyme Q10) (30 mg/day), fursultiamine (75 mg/day), and ascorbic acid (3 g/day) [31, 32]. It may appear that this treatment for mitochondrial disease improved our patient’s HbA1c.

His brain lesions almost disappeared by day 302 (Fig. 1e, f). To date, he is leading an apparently healthy life, without developing another stroke-like episode or adverse events.

To estimate the severity of his mitochondrial disease, GDF-15 in the sequentially collected sera was measured at Kurume University. GDF-15 was elevated to an immeasurably high level on day 0 (Fig. 2b), especially between 8 and 20 hours after the onset of the stroke-like episode (data not shown). Moreover, lactate-to-pyruvate ratio (Fig. 2c) and GDF-15 worsened even after his HbA1c improved.

**Fig. 3:**
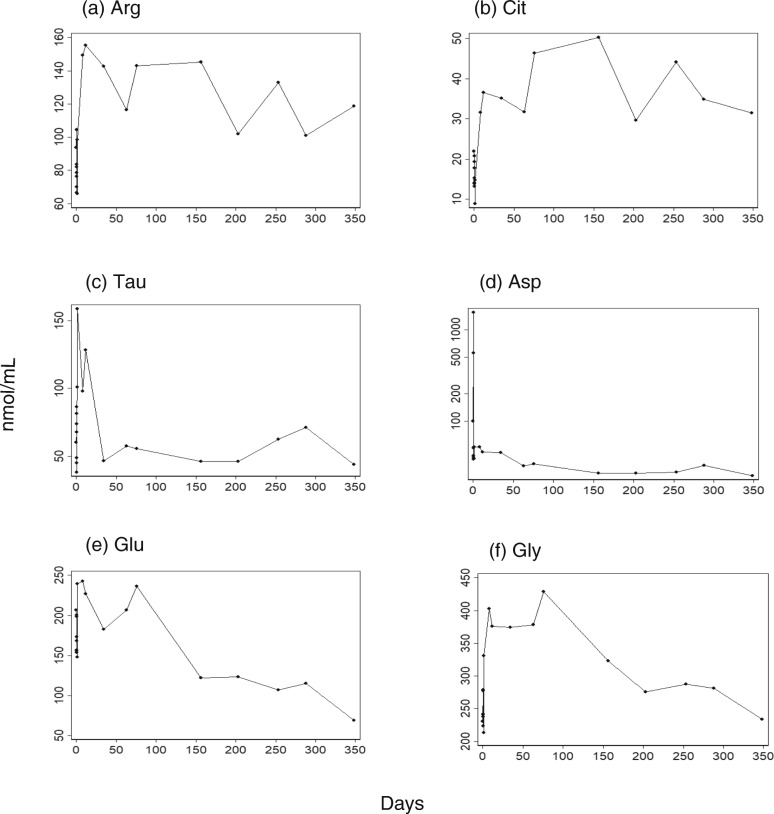
Amino acid species were measured over a year since the stroke-like episode. The measurements (nmol/mL) are presented over the days that elapsed after the stroke-like episode (day 0), for arginine (**a**), citrulline (**b**), taurine (**c**), aspartic acid (**d**), glutamic acid (**e**), and glycine (**f**). Aspartic acid (**d**) is presented with a log scale, because it exhibited an extremely high value on day 0. Amino acids were measured in serum samples, using a laboratory protocol designed for plasma samples. Therefore, no reference value is applicable. *Arg* arginine, *Asp* aspartic acid, *Cit* citrulline, *Glu* glutamic acid, *Gly* glycine, *Tau* taurine

Since mitochondrial disease has been reported to disturb amino acid metabolism [10, 12], we measured amino acids in the sera (BML, Tokyo, Japan). The laboratory protocol was designed for plasma samples. However, we used serum samples because, to the best of our knowledge, no commercial institutions measured amino acids in serum samples. Many species of amino acids, including arginine, citrulline, and taurine, exhibited a decreased level on day 0, but surged on day 1 (Fig. 3, other amino acids are presented in Additional file 1: Figure S1). We measured the amino acids at 10 time points on day 0 to confirm that this finding was not due to an erroneous value obtained from a single sampling (data not shown). The decreases in arginine and citrulline in MELAS are consistent with a previous report [12]. Remarkably, aspartic acid exhibited an extremely high value on day 0 (Fig. 3d). The derangement in the amino acid seemed to have continued after the hospital discharge.

## Discussion and conclusions

We identified an adult case of MIDD that overlapped with MELAS. Our patient had a stroke-like episode possibly triggered by DKA (or vice versa). To the best of our knowledge, this is the first report in which the longitudinal effects of L-arginine therapy on GDF-15 and multiple amino acids are presented.

During the 1-year period after this episode, his clinical symptoms, severity of diabetes, as well as MRI findings, generally improved. However, the insufficiency of mitochondrial function, as measured by GDF-15 and the lactate-to-pyruvate ratio, apparently worsened. This indicates the necessity to monitor these values continuously for a long period in a patient with mitochondrial disease, even while the symptoms appear to be improving.

Mitochondrial disease is recognized as an increasingly important etiology of diabetes mellitus in the adult population. Mitochondrial disease can cause life-threatening DKA [33] and recurrent stroke-like episodes. The intravenous administration of L-arginine is an effective treatment for stroke-like episodes of MELAS [8, 9], whereas orally administered L-arginine is preventive against stroke-like episodes [34]. In addition, numerous drugs are contraindicated in patients with mitochondrial disease [13, 15, 32, 35]. Therefore, identifying a patient with mitochondrial disease at the earliest stage is of paramount importance. However, genetic testing can provide an ambiguous diagnosis, due to the “heteroplasmy” of mitochondrial disease [6]. A negative test obtained from peripheral blood may not ensure that the vital organs are unaffected. Muscle fibers, skin, and urine sediment may represent the disease in the vital organs with a greater accuracy than peripheral blood [36]. However, to the best of our knowledge, all the commercial tests available in Japan use only the peripheral blood or muscle fiber, the latter of which requires an invasive procedure. Therefore, biomarkers are more useful for screening mitochondrial disease. In our patient, GDF-15 remained at a high level at any given time, underlining its utility as a biomarker for mitochondrial dysfunction. In particular, GDF-15 showed an abrupt increase on the day of the stroke-like episode (day 0). Therefore, GDF-15 may be useful in differentiating between a stroke-like episode and a stroke, in a patient with MIDD/MELAS.

In addition, many species of amino acids exhibited a sudden increase or decrease on the same day. This finding indicates that a stroke-like episode is a reflection of an unusual pathological process affecting the metabolism of amino acids. This derangement of amino acids was not restricted to arginine, citrulline, and taurine, but involved numerous amino acids, as was suggested previously [12]. Of note, the aspartic acid level increased dramatically within 8 hours after the stroke-like episode. Aspartic acid, as well as glutamate and glycine (Fig. 3e, f), remained elevated for several months after this episode. Since these three amino acids are neurotransmitters, these amino acids, particularly aspartic acid, may be involved in the pathogenesis of a stroke-like episode.

In the present case, L-arginine and the cocktail therapy for mitochondrial disease seemed to have improved the diabetes mellitus. However, this may be incidental, because we increased the dose of insulin when these therapies were initiated. Large-scale studies are warranted to explore the effect of L-arginine and the cocktail therapy on diabetes mellitus.

## Supplementary information

**Additional file 1: Figure S1.** The measured values (nmol/mL) are presented over the days that elapsed after the stroke-like episode (day 0), for the amino acid species that were not presented in Fig. 3.

## Data Availability

All the data obtained in this study are available on request to the corresponding author. Longitudinally collected sera can be submitted on request.

## Abbreviations

DKA: Diabetic ketoacidosis
FLAIR: Fluid-attenuated inversion recovery
GDF: Growth differentiation factor
HbA1c: Glycated hemoglobin
MELAS: Mitochondrial myopathy, encephalopathy, lactic acidosis, and stroke-like episodes
MIDD: Maternally inherited diabetes and deafness
MRI: Magnetic resonance imaging

## References

[1] Murphy R, Turnbull DM, Walker M, Hattersley AT. Clinical features, diagnosis and management of maternally inherited diabetes and deafness (MIDD) associated with the 3243A>G mitochondrial point mutation. Diabet Med. 2008;25:383–99. 10.1111/j.1464-5491.2008.02359.x 18294221

[2] Fujita Y, Ito M, Kojima T, Yatsuga S, Koga Y, Tanaka M. GDF15 is a novel biomarker to evaluate efficacy of pyruvate therapy for mitochondrial diseases. Mitochondrion. 2015;20:34–42. 10.1016/j.mito.2014.10.006 25446397

[3] Abdullah M, Vishwanath S, Elbalkhi A, Ambrus JL Jr. Mitochondrial myopathy presenting as fibromyalgia: a case report. J Med Case Rep. 2012;6:55. 10.1186/1752-1947-6-55 PMC3293012 22325469

[4] Abu-Amero KK, Al-Dhalaan H, Bohlega S, Hellani A, Taylor RW. A patient with typical clinical features of mitochondrial encephalopathy, lactic acidosis and stroke-like episodes (MELAS) but without an obvious genetic cause: a case report. J Med Case Rep. 2009;3:77. 10.1186/1752-1947-3-77 PMC2783076 19946553

[5] Yatsuga S, Fujita Y, Ishii A, Fukumoto Y, Arahata H, Kakuma T, Kojima T, Ito M, Tanaka M, Saiki R, Koga Y. Growth differentiation factor 15 as a useful biomarker for mitochondrial disorders. Ann Neurol. 2015;78:814–23. 10.1002/ana.24506 PMC5057301 26463265

[6] Gorman GS, Chinnery PF, DiMauro S, Hirano M, Koga Y, McFarland R, Suomalainen A, Thorburn DR, Zeviani M, Turnbull DM. Mitochondrial diseases. Nat Rev Dis Primers. 2016;2:16080. 10.1038/nrdp.2016.80 27775730

[7] Murakami T, Ueba Y, Shinoto Y, Koga Y, Kaneda D, Hatoko T, Kato T, Yonemitsu S, Muro S, Oki S. Successful Glycemic Control Decreases the Elevated Serum FGF21 Level without Affecting Normal Serum GDF15 Levels in a Patient with Mitochondrial Diabetes. Tohoku J Exp Med. 2016;239:89–94. 10.1620/tjem.239.89 27212224

[8] Kubota M, Sakakihara Y, Mori M, Yamagata T, Momoi-Yoshida M. Beneficial effect of L-arginine for stroke-like episode in MELAS. Brain Dev. 2004;26:481–3. discussion 480 10.1016/j.braindev.2004.01.006 15351086

[9] Koga Y, Akita Y, Nishioka J, Yatsuga S, Povalko N, Tanabe Y, Fujimoto S, Matsuishi T. L-arginine improves the symptoms of strokelike episodes in MELAS. Neurology. 2005;64:710–2. 10.1212/01.WNL.0000151976.60624.01 15728297

[10] Koga Y, Povalko N, Nishioka J, Katayama K, Kakimoto N, Matsuishi T. MELAS and L-arginine therapy: pathophysiology of stroke-like episodes. Ann N Y Acad Sci. 2010;1201:104–10. 10.1111/j.1749-6632.2010.05624.x 20649546

[11] Koga Y, Povalko N, Nishioka J, Katayama K, Yatsuga S, Matsuishi T. Molecular pathology of MELAS and L-arginine effects. Biochim Biophys Acta. 1820;2012:608–14. 10.1016/j.bbagen.2011.09.005 21944974

[12] El-Hattab AW, Hsu JW, Emrick LT, Wong LJ, Craigen WJ, Jahoor F, Scaglia F. Restoration of impaired nitric oxide production in MELAS syndrome with citrulline and arginine supplementation. Mol Genet Metab. 2012;105:607–14. 10.1016/j.ymgme.2012.01.016 PMC4093801 22325939

[13] Parikh S, Goldstein A, Koenig MK, Scaglia F, Enns GM, Saneto R, Anselm I, Cohen BH, Falk MJ, Greene C, *et al.* Diagnosis and management of mitochondrial disease: a consensus statement from the Mitochondrial Medicine Society. Genet Med. 2015;17:689–701. 10.1038/gim.2014.177 PMC5000852 25503498

[14] Koenig MK, Emrick L, Karaa A, Korson M, Scaglia F, Parikh S, Goldstein A. Recommendations for the Management of Strokelike Episodes in Patients With Mitochondrial Encephalomyopathy, Lactic Acidosis, and Strokelike Episodes. JAMA Neurol. 2016;73:591–4. 10.1001/jamaneurol.2015.5072 26954033

[15] Parikh S, Goldstein A, Karaa A, Koenig MK, Anselm I, Brunel-Guitton C, Christodoulou J, Cohen BH, Dimmock D, Enns GM, *et al.* Patient care standards for primary mitochondrial disease: a consensus statement from the Mitochondrial Medicine Society. Genet Med. 2017;19 10.1038/gim.2017.107. PMC7804217 28749475

[16] El-Hattab AW, Emrick LT, Hsu JW, Chanprasert S, Almannai M, Craigen WJ, Jahoor F, Scaglia F. Impaired nitric oxide production in children with MELAS syndrome and the effect of arginine and citrulline supplementation. Mol Genet Metab. 2016;117:407–12. 10.1016/j.ymgme.2016.01.010 PMC4818739 26851065

[17] Suzuki T, Nagao A. Human mitochondrial diseases caused by lack of taurine modification in mitochondrial tRNAs. Wiley Interdiscip Rev RNA. 2011;2:376–86. 10.1002/wrna.65 21957023

[18] Ohsawa Y, Hagiwara H, Nishimatsu SI, Hirakawa A, Kamimura N, Ohtsubo H, Fukai Y, Murakami T, Koga Y, Goto YI, *et al.* Taurine supplementation for prevention of stroke-like episodes in MELAS: a multicentre, open-label, 52-week phase III trial. J Neurol Neurosurg Psychiatry. 2019;90:529–36. 10.1136/jnnp-2018-317964 PMC6581075 29666206

[19] Kolb SJ, Costello F, Lee AG, White M, Wong S, Schwartz ED, Messe SR, Ellenbogen J, Kasner SE, Galetta SL. Distinguishing ischemic stroke from the stroke-like lesions of MELAS using apparent diffusion coefficient mapping. J Neurol Sci. 2003;216:11–5. 10.1016/s0022-510x(03)00218-1 14607297

[20] Cai SS, von Coelln R, Kouo TJ. Migratory stroke-like lesions in a case of adult-onset mitochondrial encephalomyopathy, lactic acidosis, and stroke-like episodes (MELAS) syndrome and a review of imaging findings. Radiol Case Rep. 2016;11:425–9. 10.1016/j.radcr.2016.09.003 PMC5128397 27920873

[21] Tetsuka S, Tagawa A, Ogawa T, Otsuka M, Hashimoto R, Kato H. Importance of Distinguishing Between Mitochondrial Encephalomyopathy With Elderly Onset of Stroke-Like Episodes and Cerebral Infarction. J Clin Med Res. 2017;9:812–9. 10.14740/jocmr3122w PMC5544489 28811861

[22] Nakamura S, Yoshinari M, Wakisaka M, Kodera H, Doi Y, Yoshizumi H, Asano T, Iwase M, Mihara F, Fujishima M. Ketoacidosis accompanied by epileptic seizures in a patient with diabetes mellitus and mitochondrial myopathy, encephalopathy, lactic acidosis and stroke-like episodes (MELAS). Diabetes Metab. 2000;26:407–10. 11119021

[23] Yatsuga S, Povalko N, Nishioka J, Katayama K, Kakimoto N, Matsuishi T, Kakuma T, Koga Y. MELAS: a nationwide prospective cohort study of 96 patients in Japan. Biochim Biophys Acta. 1820;2012:619–24. 10.1016/j.bbagen.2011.03.015 21443929

[24] Saisho Y, Kou K, Tanaka K, Abe T, Kurosawa H, Shimada A, Meguro S, Kawai T, Itoh H. Postprandial serum C-peptide to plasma glucose ratio as a predictor of subsequent insulin treatment in patients with type 2 diabetes. Endocr J. 2011;58:315–22. 10.1507/endocrj.k10e-399 21415555

[25] Okuno Y, Komada H, Sakaguchi K, Nakamura T, Hashimoto N, Hirota Y, Ogawa W, Seino S. Postprandial serum C-peptide to plasma glucose concentration ratio correlates with oral glucose tolerance test- and glucose clamp-based disposition indexes. Metabolism. 2013;62:1470–6. 10.1016/j.metabol.2013.05.022 23831440

[26] Goto Y, Nonaka I, Horai S. A mutation in the tRNA^(Leu)(UUR)^ gene associated with the MELAS subgroup of mitochondrial encephalomyopathies. Nature. 1990;348:651–3. 10.1038/348651a0 2102678

[27] van den Ouweland JM, Lemkes HH, Ruitenbeek W, Sandkuijl LA, de Vijlder MF, Struyvenberg PA, van de Kamp JJ, Maassen JA. Mutation in mitochondrial tRNA^(Leu)(UUR)^ gene in a large pedigree with maternally transmitted type II diabetes mellitus and deafness. Nat Genet. 1992;1:368–71. 10.1038/ng0892-368 1284550

[28] Reardon W, Ross RJ, Sweeney MG, Luxon LM, Pembrey ME, Harding AE, Trembath RC. Diabetes mellitus associated with a pathogenic point mutation in mitochondrial DNA. Lancet. 1992;340:1376–9. 10.1016/0140-6736(92)92560-3 1360090

[29] Kadowaki H, Tobe K, Mori Y, Sakura H, Sakuta R, Nonaka I, Hagura R, Yazaki Y, Akanuma Y, Kadowaki T. Mitochondrial gene mutation and insulin-deficient type of diabetes mellitus. Lancet. 1993;341:893–4. 10.1016/0140-6736(93)93101-6 8096591

[30] Nesbitt V, Pitceathly RD, Turnbull DM, Taylor RW, Sweeney MG, Mudanohwo EE, Rahman S, Hanna MG, McFarland R. The UK MRC Mitochondrial Disease Patient Cohort Study: clinical phenotypes associated with the m.3243A>G mutation--implications for diagnosis and management. J Neurol Neurosurg Psychiatry. 2013;84:936–8. 10.1136/jnnp-2012-303528 23355809

[31] Omata T, Fujii K, Takanashi J, Murayama K, Takayanagi M, Muta K, Kodama K, Iida Y, Watanabe Y, Shimojo N. Drugs indicated for mitochondrial dysfunction as treatments for acute encephalopathy with onset of febrile convulsive status epileptics. J Neurol Sci. 2016;360:57–60. 10.1016/j.jns.2015.11.043 26723974

[32] The Japanse Society of Mitochondrial Research and Medicine. Mitochondria disease management manual 2017 [in Japanese]. Tokyo: Shindan to chiryou sha; 2017.

[33] Kadowaki T, Kadowaki H, Mori Y, Tobe K, Sakuta R, Suzuki Y, Tanabe Y, Sakura H, Awata T, Goto Y, *et al.* A subtype of diabetes mellitus associated with a mutation of mitochondrial DNA. N Engl J Med. 1994;330:962–8. 10.1056/NEJM199404073301403 8121460

[34] Koga Y, Povalko N, Inoue E, Nakamura H, Ishii A, Suzuki Y, Yoneda M, Kanda F, Kubota M, Okada H, Fujii K. Therapeutic regimen of L-arginine for MELAS: 9-year, prospective, multicenter, clinical research. J Neurol. 2018;265:2861–74. 10.1007/s00415-018-9057-7 PMC6244654 30269300

[35] Parikh S, Saneto R, Falk MJ, Anselm I, Cohen BH, Haas R, Medicine Society TM. A modern approach to the treatment of mitochondrial disease. Curr Treat Options Neurol. 2009;11:414–30. 10.1007/s11940-009-0046-0 PMC3561461 19891905

[36] Zhiping W, Quwen L, Hai Z, Jian Z, Peiyi G. Application of molecular imaging combined with genetic screening in diagnosing MELAS, diabetes and recurrent pancreatitis. Folia Neuropathol. 2016;54:66–71. 10.5114/fn.2016.58917 27179223

